# Many ABCs make light work with cholesterol

**DOI:** 10.18632/aging.100596

**Published:** 2013-08-31

**Authors:** Dmitri Sviridov

**Affiliations:** Baker IDI Heart and Diabetes Institute, Melbourne, Australia

Cholesterol-centric model of the Universe is gaining recognition. The key role of cholesterol metabolism in cardiovascular diseases has been accepted long ago; emerging evidence now supports the central role of cholesterol metabolism in neurodegenerative, metabolic, skin and infectious diseases. Cellular cholesterol content has to be maintained within tight limits, or else; not surprisingly, therefore, a number of regulatory mechanisms provide multi-layer protection of cholesterol homeostasis. Deficiency of cholesterol is rarely an issue: all human nucleated cells have efficient cholesterol biosynthesis machinery capable of satisfying the need in cholesterol under almost any circumstance. Excess of cholesterol is a different matter, it almost inevitably causes severe pathology with clinical manifestation depending on the tissue affected. Difficulty is withstanding excessive cholesterol is because the only cell type capable of degrading cholesterol is hepatocyte; other cells and tissues have to find a way around it. Reducing production of cholesterol and its uptake from lipoproteins provide some relief, but can only go so far. Esterification of cholesterol, although reduces toxicity, is nevertheless a trap as reversing it (hydrolysis of cholesteryl esters) is a very slow process. The most important regulatory pathway in maintaining cholesterol homeostasis is therefore reverse cholesterol transport (RCT).

The essence of RCT is in taking excessive cholesterol from any cell of the body and transporting it through the blood to the liver and intestine where it can be degraded and/or secreted. Cholesterol has limited aqueous solubility and in the blood it has to be carried by lipoproteins. A lipoprotein that takes cholesterol from cells and carries it to the liver/intestine is high density lipoprotein (HDL)/apolipoprotein A-I (apoA-I). Although limited amount of cholesterol can passively diffuse from cells to lipoproteins, most of excessive cholesterol is released in energy-dependent and controlled manner, via a process termed cholesterol efflux. Several transporters control cholesterol efflux, but the most important is ATP binding cassette transporter A1 (ABCA1). ABCA1 interacts with extracellular apoA-I loading it with cellular phospholipids and cholesterol; by doing so it transforms apoA-I into nascent HDL and relieves cell of excessive cholesterol. The rate of the efflux depends on the abundance of ABCA1 and its functionality, both are regulated on several levels. On transcriptional level ABCA1 is regulated by the Liver X Receptor (LXR), a nuclear receptor capable of stimulation transcription of ABCA1 gene when bound to an agonist. On post-transcriptional level abundance of ABCA1 is regulated through its degradation in both lysosomes and proteasomes as well as through action of calpain. Functionality of ABCA1 is regulated through its phosphorylation and trafficking to and from plasma membrane. Different levels of regulation are interconnected; for example removal of ABCA1 from plasma membrane reduces its functionality, but also leads to degradation. Hozoji-Inada et al. have recently proposed that LXR regulates ABCA1 on both transcriptional and post-translational levels [1]. They suggested that one isoform of LXR, LXRβ, binds to ABCA1 preventing ATP hydrolysis and shutting down its function. LXR agonist disrupts this complex, on the one hand, restoring ABCA1 functionality and on the other, allowing LXRβ to travel to the nucleus and to initiate transcription of the ABCA1 gene. We have recently found another player in this game [2].

ABCA12 is known for its role in maintaining skin barrier function. Deficiency in ABCA12 is the cause of Harlequin ichthyosis, an often fatal skin disease. We however noticed that fibroblasts from ABCA12^−/−^ mouse are extremely susceptible to challenge with excessive cholesterol [3]. Mechanistic studies on macrophages demonstrated that ABCA12 deficient cells fail to respond to activation with LXR agonist. Interestingly, expression of the ABCA1 gene was properly stimulated, but increases in ABCA1 protein abundance were blunted and cholesterol efflux was not stimulated at all. Another unexpected effect of ABCA12 deficiency was a fall in abundance of LXRβ; overexpression of LXRβ reversed the effects of ABCA12 deficiency. Like Hozoji-Inada we found that LXRβ binds to ABCA1, ABCA12 binds to both LXRβ and ABCA1, and while in ABCA12^+/+^ cells LXR dissociates when agonist is added, this didn't happen in ABCA12^−/−^ cells. In vivo, when apoe^−/−^ mice were transplanted with apoe^−/−/^ Abca12^−/−^ bone marrow, this lead to impairment of reverse cholesterol transport and significant acceleration of development of atherosclerosis. Our conclusion is that ABCA12, along with LXRβ, is a part of a regulatory complex controlling ABCA1 functionality.

Reverse cholesterol transport and specifically ABCA1 are involved in many diseases that are common in middle to late ages, most importantly in type 2 diabetes [4] and Alzheimer disease [5]. And yet, epidemiological data connecting ABCA1 polymorphism and susceptibility to these diseases are not very convincing [6]. It is conceivable that mutations in proteins within pathways regulating ABCA1 functionality (such as LXRβ or ABCA12) are more important for the outcomes than polymorphism of ABCA1 itself. On the other hand, the elements of the regulatory pathways may present many potential targets for therapeutic interventions.
